# Neurosarcoidosis presenting as CRVO combined CRAO: a biopsy-proven case report of a Chinese patient

**DOI:** 10.1186/s12886-020-01624-5

**Published:** 2020-08-27

**Authors:** Chaoyi Feng, Qian Chen, Wei Liu, Yingwen Bi, Jiang Qian, Min Wang, Xinghuai Sun, Guohong Tian

**Affiliations:** 1 Department of Ophthalmology, Eye Ear Nose and Throat Hospital, Fudan University, 83 Fenyang Road, Shanghai, 200031 China; 2 Department of Pathology, Eye Ear Nose and Throat Hospital, Fudan University, Shanghai, 200031 China; 3 NHC Key Laboratory of Myopia (Fudan University), Key Laboratory of Visual Impairment and Restoration, Shanghai, China

**Keywords:** Optic neuropathy, Neurosarcoidosis, Retinal vein occlusion, Optic nerve biopsy

## Abstract

**Background:**

Neurosarcoidosis is a rare systemic disorder that can affect the eye and other organs, including the central nervous system. Neurosarcoidosis infiltrating the optic nerve presenting as central retinal vein occlusion combined with artery ischaemia has not been reported in the literature previously. We describe a Chinese patient presenting with acute monocular vision loss, in whom an optic nerve biopsy confirmed the diagnosis of neurosarcoidosis.

**Case presentation:**

A 47-year-old woman complained of acute decreased vision in her left eye over the course of 1 month. She reported that her vision deteriorated quickly within first 3 days of consulting an ophthalmologist at a local hospital. She was diagnosed with central retinal vein occlusion after funduscopic examination and fundus fluorescein angiography, and the vision in her left eye further deteriorated to no light perception. An orbital magnetic resonance imaging showed an abnormal T1-weighted image of the optic nerve after contrast enhancement. She was referred to a neuro-ophthalmologist for further evaluation. After routine blood tests ruled out infectious and metastatic diseases, she was prescribed 500 mg/d methylprednisolone for 5 days, but her vision did not improve. As she could still not perceive light, an optic nerve biopsy was performed, and the histopathology revealed non-necrotising granuloma that was consistent with neurosarcoidosis.

**Conclusions:**

Isolated optic nerve infiltration by neurosarcoidosis without the involvement of the central nervous system or other systemic organs is challenging to diagnose. Biopsy of the optic nerve sheath is crucial for the final diagnosis of neurosarcoidosis. Therefore, a comprehensive ophthalmologic and systemic examination and work-up for inflammation of the eye, chest, and central nervous system should be conducted for atypical cases.

## Background

Neurosarcoidosis is very rare, but the most common neuro-ophthalmic manifestation is optic neuropathy [1–3]. Diagnosing neurosarcoidosis without the involvement of other organs is very challenging. Due to its unspecific clinical manifestation and laboratory findings, neurosarcoidosis can masquerade as optic neuritis, optic nerve sheath meningioma, a metastatic tumour, or a variety of other issues [4–6].

We describe a Chinese middle-aged woman who presented with monocular central retinal vein occlusion (CRVO) combined with retinal artery ischaemia. An optic nerve biopsy confirmed the diagnosis of neurosarcoidosis. To our knowledge, this is the first case report of neurosarcoidosis infiltrating the optic nerve presenting with CRVO combined central retinal artery occlusion (CRAO).

## Case presentation

**Fig. 1:**
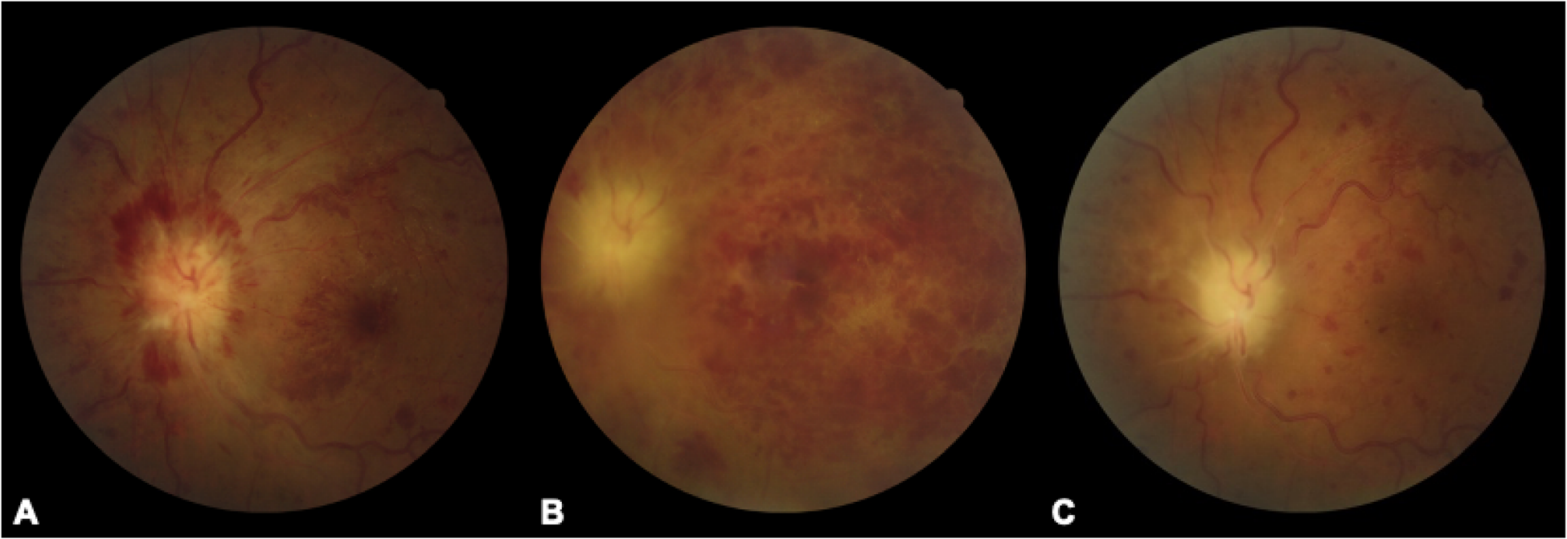
Fundus photographs of the patient’s left eye. **a**: At presentation, the optic disc showed severe swelling with peripapillary and posterior retinal haemorrhaging. The macula also showed exudation and haemorrhaging. **b**: A central retinal vein occlusion with optic edema, dilated and tortuous veins, and extensive intraretinal haemorrhage. **c**: After methylprednisolone treatment, the optic disc swelling resolved along with residual retinal haemorrhaging with the narrowing of the vessels

**Fig. 2:**
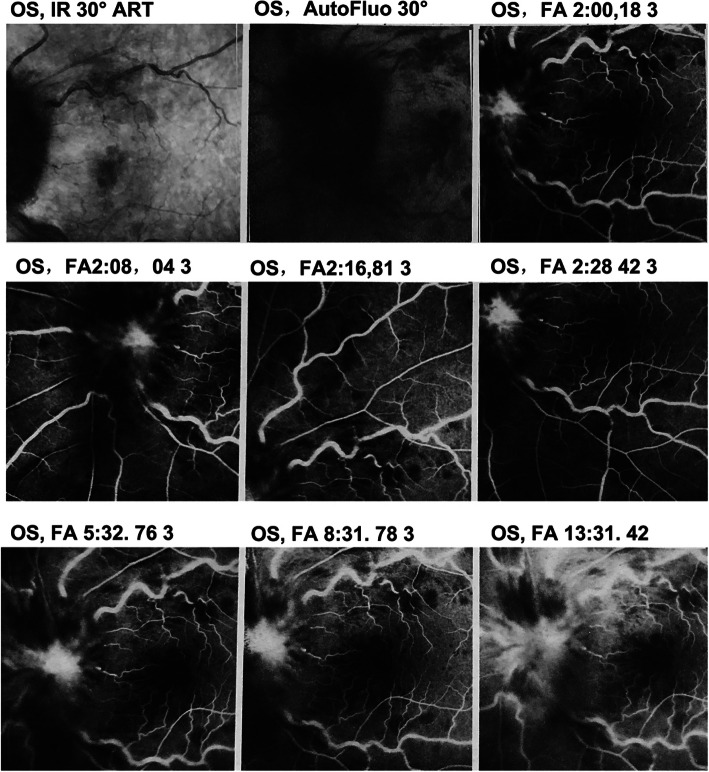
Fundus fluorescein angiography showing the tortuous retinal veins, optic oedema, and optic disc leakage at the late stage of the left eye

A 47-year-old Chinese woman complained of blurred monocular vision in her left eye 1 month prior to her visit. She also reported that her vision deteriorated very quickly and that she became almost blind after 3 days since the onset of the disease. She denied headache, fever, or cough prior to the vision problem. The ophthalmologist at the local hospital found the visual acuity was 20/20 in the right eye with suspicious light perception in the left eye. The fundus examine (Fig. 1a) and fundus fluorescein angiography (Fig. 2) were performed. She was diagnosed with CRVO in her left eye and was followed up for 1 month. The patient continued to lose vision, and the swelling of the optic disc and retina did not improve. She was referred to a neuro-ophthalmologist for further evaluation. She was a considerably healthy housewife living in a southeastern city of China. The chart review was only remarkable for mild hypertension. She neither smoked nor drank alcohol. There was no family history of neurological or hereditary diseases.

The neuro-ophthalmological examination revealed the patient to be alert and oriented. Her visual acuity was 20/20 in the right eye with no light perception in the left eye. The left pupil showed no light reaction and an afferent pupillary defect. Slit lamp examination revealed no keratic precipitates or cells in the vitreous of either eye. The intraocular pressure was 13 mmHg in the right eye and 11 mmHg in the left eye. Funduscopic examination revealed a central retinal vein occlusion with optic oedema, dilated and tortuous veins, and extensive intraretinal haemorrhage. (Fig. 1b). The right fundus was unremarkable. There were no other abnormal focal neurological signs.

Routine laboratory tests, including complete blood count and liver and renal function, were unremarkable. A rheumatology panel, including erythrocyte sedimentation ratio, C-reactive protein, anti-nuclear antibody, anti-extractable nuclear antibodies, and anti-neutrophil cytoplasmic antibody, were unremarkable. Angiotensin converting enzyme levels were within the normal limit. An infectious disease panel, including human immunodeficiency virus, herpes simplex virus, cytomegalovirus, *Treponema pallidum* antibody, and T-spot, were negative.

**Fig. 3:**
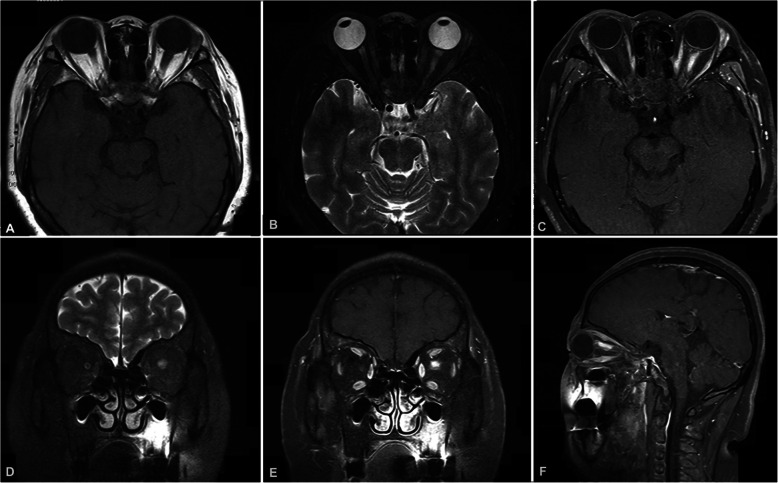
The orbital fat-suppressed T1-weighted (T1W) magnetic resonance imaging showing (**a**): the enlargement of the left optic nerve compared with the right; (**b**): the left optic nerve showing hyperintensity on T2-weighted (T2W) imaging; (**c**): the dramatic enhancement of the anterior orbital portion of the left optic nerve dramatically enhanced after gadolinium injection; (**d**): coronal T2W imaging; (e): coronal T1W imaging after contrast; (**f**): sagittal T1W imaging after contrast showing segmental enhancement of the left optic nerve with a distinct boundary

The brain and orbital magnetic resonance imaging (MRI) with contrast showed enlargement of the left optic nerve in the orbit. The anterior part of the optic nerve showed heterogenous enhancement. There were no other brain lesions or masses (Fig. 3). Chest computed tomography (CT) with contrast was unremarkable. The lumbar puncture showed normal intracranial pressure with normal cerebrospinal fluid components and biochemistry.

**Fig. 4:**
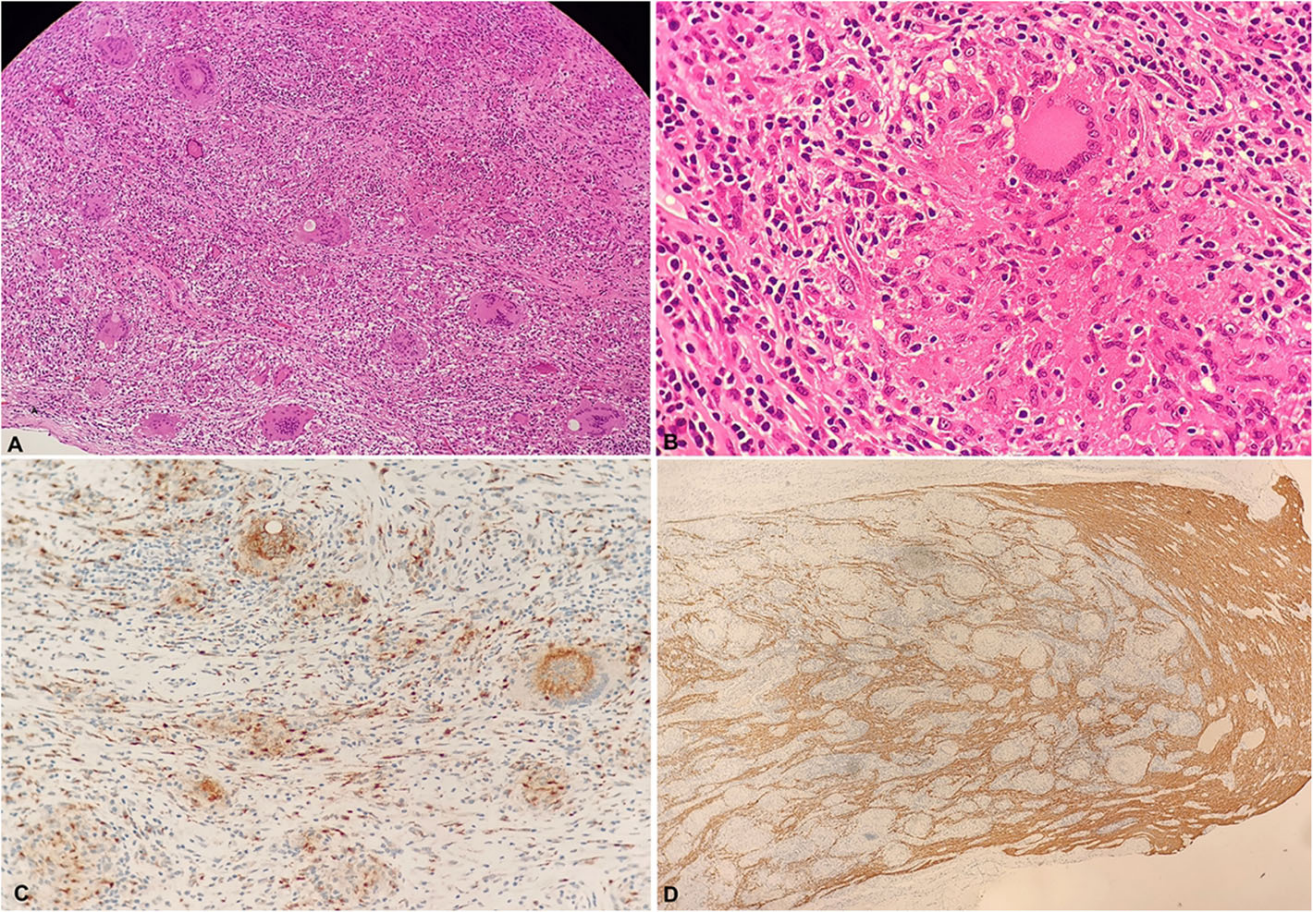
The left optic nerve biopsy showing the optic nerve tissues. **a**: The normal structure of the optic nerve was disrupted with numerous non-caseating granulomas (haematoxylin-eosin (HE) stain, × 40). **b**: A giant cell with multiple nuclei is surrounded by a rim composed of lymphoid cells and fibrotic collagen (HE stain, × 400). Immunohistochemistry was negative for CD68 (**c**, × 200) and showed a loss of GFAP (**d**, × 40)

She was diagnosed with optic neuropathy combined with CRVO/CRAO of unknown causes. She was given 500 mg/d intravenous methylprednisolone for 5 days and was then tapered off. Her visual acuity in the left eye maintained no light perception after treatment and the optic disc swelling resolved along with residual retinal haemorrhaging with the narrowing of the vessels. (Fig. 1c). The macular optical coherence tomography showed thinning of the inner layer of the right retina due to retinal artery occlusion. As she could not perceive light in her left eye, she was scheduled for an optic nerve biopsy. Histopathology showed that the optic nerve had lost its original structure and was infiltrated with numerous inflammatory non-caseating granulomas, which was consistent with sarcoidosis (Fig. 4). The final diagnosis of neurosarcoidosis was made according to the clinical manifestation and pathology of the patient. She was only seen for follow-up with no further treatment due to no other systemic involvement of sarcoidosis. Her left eye maintained no light perception and the retina thinned afterwards.

## Discussion and conclusions

Sarcoidosis is a systematic disorder that is characterised by inflammatory non-caseating granulomas in multiple organs, commonly involving the lungs, eyes, and skin [7]. In Japan, the annual incidence ranges from 1 to 2 cases per 100,000 people compared to the worldwide incidence of 10–20 cases per 100,000 people [8]. Neurosarcoidosis is a very rare disease that affects about 5–10% of sarcoidosis patients but can be found in more than 25% of autopsies [2, 3]. The most commonly reported presenting manifestation of neurosarcoidosis is cranial neuropathy and the most frequently affected cranial nerves are the optic and facial nerves, according to the literature [9–11].

The diagnosis of neurosarcoidosis is very challenging when lesions are localised in the central nervous system (CNS) and with no other organs involved, such as in the case we have described. Neurosarcoidosis is even more infrequently diagnosed in Chinese populations due to the low incidence of sarcoidosis in people of Asian descent, in addition to fewer tissue biopsies performed.

Our patient had neurosarcoidosis presenting as a common CRVO combined with retinal artery ischaemia and poor vision function, which has not been reported before. The granulomatous swollen appearance of the optic disc with no other signs of uveitis made it difficult to differentiate an infiltration from metastatic disease. Further MRI findings of the optic nerve enlargement indicated that the original optic neuropathy led to the occlusion of both the retrobulbar central retinal artery and vein. This explained why our patient responded poorly to a high-dose steroid treatment. As she could not perceive light, an optic nerve biopsy was performed and the final diagnosis was made.

Although a rare disease, neurosarcoidosis might be underdiagnosed in some patients with isolated CNS involvement, which is very challenging to biopsy. Due to quick responses to steroids, many patients with sarcoidosis infiltrating the optic nerve were misdiagnosed with recurrent optic neuritis and were put on immunosuppressive agents. Although the treatment is very effective, but it might cover up the systemic symptoms of sarcoidosis. MRI features can be helpful to differentiate neurosarcoidosis from other diseases, such as meningioma, schwannomas, lymphomas, or metastatic conditions [12–14]. In our experience, the discrete features (like the optic and cranial nerves, spinal cord, leptomeninges, and dura mater), rather than a contiguous enhancement of the lesions, will be helpful for differential diagnosis.

In conclusion, the diagnosis of isolated neurosarcoidosis localised in the CNS is challenging. Comprehensive ophthalmologic and systemic examinations and work-up for inflammation of the eye, chest, and CNS are important. Tissue biopsy is crucial for final diagnosis of atypical cases and will deepen the understanding of the disease mechanism.

## Data Availability

Almost all data generated or analysed during this study are included in this published article, and the other datasets used or analysed during the current study will be made available from the corresponding author upon reasonable request.

## Abbreviations

MRI: Magnetic resonance imaging
CRVO: Central retinal vein occlusion
CT: Computed tomography
CRAO: Central retinal artery occlusion
NLP: No light perception
OCT: Optical coherence tomography
CNS: Central nervous system
